# Camptocormia as a feature of Mc Ardle's disease: A case report

**DOI:** 10.1016/j.ymgmr.2025.101197

**Published:** 2025-02-01

**Authors:** Mathilde Nicolas, Chloé Giret, Sybille Pellieux, Annick Toutain, Anne-Marie Bergemer-Fouquet, Pascal Laforêt, Loic Bouilleau, François Maillot

**Affiliations:** a CHRU de Tours, Service de Médecine Interne, Centre de Référence des Maladies Héréditaires du Métabolisme Tours, France; b Université de Tours, France; c CHRU de Tours, Service de Médecine Physique et Réadaptation, Tours, France; d CHRU de Tours, Service de Génétique, Tours, France; e CHRU de Tours, Service d'Anatomopathologie, Tours, France; f UMR INSERM 1253 “iBraiN”, Tours, France; g CHRU de Tours, Service de Radiologie, Tours, France; h Université de Paris Saclay, Paris, France; i APHP Hôpital Raymond Poincaré, Service de Neurologie, Garches, France

**Keywords:** Glycogen storage disease type 5, McArdle's disease, Camptocormia

## Abstract

Glycogen storage disease type 5 (GSD) is an autosomal recessive metabolic myopathy caused by pathogenic variants in the *PYGM* gene. We report the case of a patient with typical exercise intolerance with a “second wind” phenomenon, associated with camptocormia which is not commonly recognized as a feature of the disease. Molecular analysis of the *PYGM* gene the common c.148C > T [p.(Arg50*)] variant and a missense variant in exon 12, c.1471C > T [p.(Arg491Cys)]. GSD 5 and Pompe disease are both glycogen storage diseases in which axial involvement has been described. Although probably underestimated, severe axial myopathy has been rarely reported in GSD 5. We suggest that the long-lasting symptoms associated with camptocormia should be considered as possible initial features of GSD 5.

## 1 Introduction

Glycogen storage disease type 5 (GSD 5, Mc Ardle's disease) is an autosomal recessive metabolic myopathy caused by pathogenic variants in the *PYGM* gene leading to myophosphorylase deficiency [1,2]. Patients usually present at adolescence with exercise intolerance, myalgia, early fatigue, muscle weakness and acute rhabdomyolysis episodes [2]. Symptoms related to physical exercise are predominant, but permanent weakness may occur, usually at an advanced stage of life [3]. In rare cases, patients are poorly symptomatic or present atypical symptoms, leading to a late diagnosis [2,4].

Camptocormia, also known as “bent spine syndrome”, is also a rare entity characterized by an anterior flexion of the thoracolumbar spine, worsened by prolonged standing or walking, which can be corrected by passive extension of the spine. Camptocormia can be associated with a large spectrum of musculoskeletal and neurological conditions. Neuromuscular etiologies of camptocormia include inflammatory myopathies, muscular dystrophies, late onset myotonic myopathies as well as endocrine and metabolic myopathies [5]. To date, camptocormia related to metabolic myopathies have been described in GSD 5 [[6], [7], [8]] and Pompe disease [9,10]. In the present report, we describe a case of camptocormia revealing GDS 5.

## 2 Case report

**Fig. 1:**
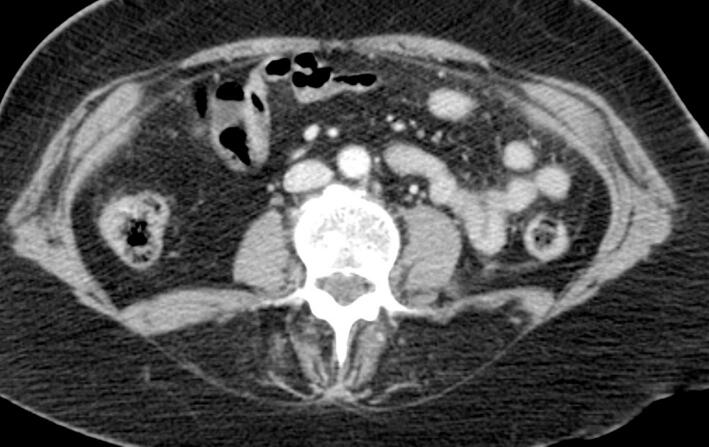
Massive fat infiltration of the lumbar spinal extensors. Respect for the lumbar and psoas squares.

**Fig. 2:**
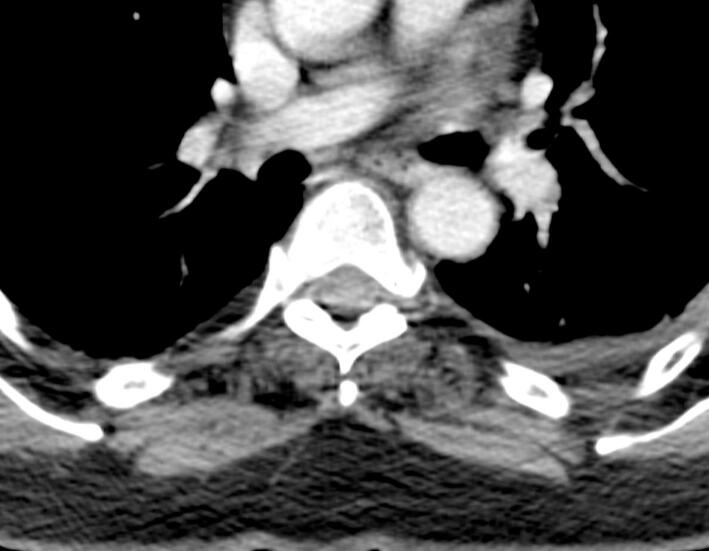
Intermediate grade fat infiltration of the iliocostalis muscles. Respect for the rhomboid and lower trapezius muscles.

**Fig. 3:**
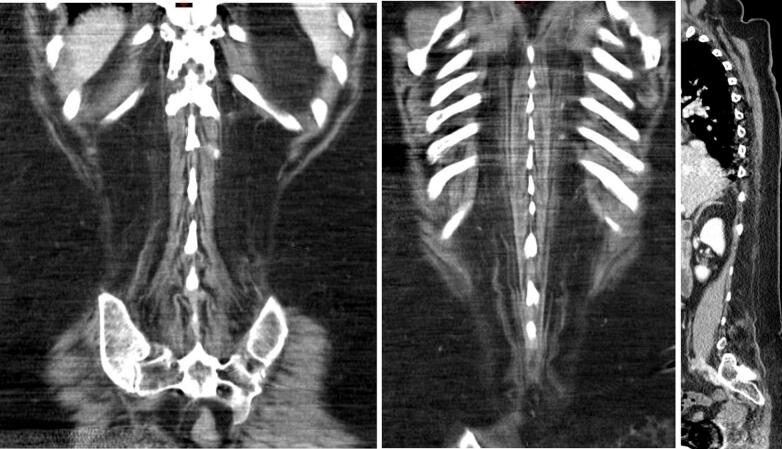
Marked bilateral and symmetrical fatty infiltration of the dorsal and lumbar spinal extensor muscles (ilio costalis and longissimus muscles).

A physically active 59y-old woman was referred to our center as she complained of exercise intolerance. This symptom has worsened over the last 7–8 years, resulting in physical disability and poor quality of life. She also reported episodes of “dark urine” following unusually intense physical activity. Her walking ability was limited to 50 m and she described a typical “second wind” phenomenon. The patient also suffered from progressive back pain and a lumbar kyphosis which developed before the age of 35y. Physical examination showed a waddling gait, symmetrical proximal weakness graded 3–4 involving the pelvic girdle muscles and hip flexors. The pelvic girdle muscles were retracted. Lower limb muscle strength was normal, except for the ankle dorsiflexors (graded 4). Symmetrical arm weakness was observed, involving the shoulder and waist and proximal arm (graded 3). Distal muscle weakness was noted in both upper limbs along with mild atrophy of the intrinsic hand muscles. The patient also had typical camptocormia associated with a deficit of the extensor muscles of the spine. These clinical features were associated with a moderate abdominal muscle deficit. Plasma creatine kinase (CK) level was 5550 U/L (*N* < 170). Electroneuromyography showed a myogenic pattern in all four limbs. Non ischemic forearm grip test was not performed. A CT scan of the paraspinal muscles (T9 and L3 levels) showed heterogeneous, diffuse and symmetrical fat replacement of the lombar spinal extensors (Fig. 1, Fig. 3) and to a lesser extent of the other paraspinal muscles. Fig. 2 shows intermediate grade fat infiltration of the iliocostalis muscles. The psoas, the rhomboid, the lower trapezius muscles and abdominal muscles showed no atrophy (Fig. 1, Fig. 2, Fig. 3). The spinal canal was normal. A muscle biopsy showed a negative strain test for myophosphorylase. Molecular analysis of the *PYGM* gene revealed two pathogenic variants, c.148C > T [p.(Arg50*)] variant and a missense variant in exon 12, c.1471C > T [p.(Arg491Cys)].

Ingestion of 1 g/kg oral sucrose before exercise increased the physical capacity of the patient and abolished episodes of myoglobinuria. Plasma CK levels decreased to 990 IU/L. Physiotherapy initially consisted of stretching exercises, postural exercises and exercises to combat retractions of the legs, as well as extension and support exercises for the trunk. A corset was made to enable the patient to stand for at least four hours a day, to relieve back pain and to help with walking. After the passive exercises, aerobic and dynamic exercises with oral sucrose ingestion were recommended depending on the patient's tolerance. At follow-up, the patient described improved exercise tolerance and walking capacity.

## 3 Discussion

The present case report suggests that a late form of GSD 5 may present with camptocormia which is not commonly recognized as a classic feature or a complication of the disease. To date, only 3 cases have been reported in the literature. The first case was a 61y-old man diagnosed with GSD 5 associated with the c.148C > T [p.(Arg50*)] and c.1948C > T [p.(Arg650*)] variants in the *PYGM* gene [6]. The second case was a 79y-old man carrying an homozygous variant c.123_124insCC [p.(Lys42Profs*48)] [7]. The last case has been recently reported and described a 78y patient carrying c.280 > T [p.(Arg94Trp)] and c.2056G > A [p.(Gly686Arg)] variants [8]. Our patient also carried the common c.148C > T [p.(Arg50*)] variant but also a c.1471C > T [p.(Arg491Cys)] variant which has already been reported only once in the literature [11]. As these four cases were genetically heterogenous, it seems difficult to make any genotype – phenotype relation to explain the onset of camptocormia. However, such reports change the classic clinical picture of GSD 5. Indeed, spinal muscles involvement is still an underrecognized feature that can be clinically obvious (as described in our case report) or detected by imaging at a subclinical stage of the disease [12]. In a study including nine patients with GSD 5, MRI imaging showed low density and heterogeneous fat replacement in the paraspinal muscles [13]. In this study, some patients have a significant fraction of muscle replaced by fat, even though they were able to maintain near-normal physical activity throughout their lives. Only one patient of these series had lumbar muscle weakness associated with a higher rate of fat replacement, probably related to age. At whole body muscles MRI scans, a homogeneous and symmetrical fatty substitution as well as a degeneration of axial muscles was observed, with predominant damage in the erector spinae over the rotator muscles. Such picture of muscle damage could explain a bent posture in some GSD 5 patients.

From the case of our patient and the previously reported cases, it seems that camptocormia is associated with late onset forms of GSD 5. Factors which may explain some late onset cases of GSD5 have not been clearly identified to date. Angiotensin-converting enzyme (*ACE*) polymorphisms have been identified as potential modulators of GSD 5 phenotype [14]. As a consequence, it has been hypothesized recently that prolonged intake of ACE inhibitors could delay the onset of GSD 5 [8]. Such hypothesis could not be confirmed in our patient as she was not treated with ACE inhibitors before her symptoms onset.

The initial step in the management of camptocormia is the detection of the underlying cause, because treatment of secondary camptocormia depends on the variety of disorder affecting paraspinal muscles [15,16]. Treatable causes of camptocormia are inflammatory myopathies, hypothyroid myopathy and osteomalacia but most often, treatment is symptomatic. As late onset GDS 5 appears to be a possible etiology of camptocormia, some specific measures may be prescribed in affected patients [17]. Some studies have shown continued positive effects after a long course of GSD 5 of gradual dynamic habituation exercises combined with carbohydrate ingestion [18]. In the case of our patient, passive exercises combined with the prior ingestion of sucrose were considered as beneficial for muscle pain. As a result, the patient reported a significant improvement in activities of daily living. However, the weakness of the spinal extensor muscles worsened. It is unclear whether our patient had a higher fat replacement rate due to age, idiopathic camptocormia or increased disease severity. In the literature, we have not found any dissociated clinical course in patients with GSD 5, although such course characteristics have been described in camptocormia in association with inflammatory myopathy or amyloid myopathy [15].

In summary, camptocormia appears to be a rare condition associated with late onset forms of GSD 5. We suggest that older patients with camptocormia should be investigated for GSD 5.

## CRediT authorship contribution statement

**Mathilde Nicolas:** Writing – original draft, Writing – review & editing. **Chloé Giret:** Conceptualization, Writing – review & editing. **Sybille Pellieux:** Validation, Visualization, Writing – review & editing. **Annick Toutain:** Supervision, Validation, Writing – review & editing. **Anne-Marie Bergemer-Fouquet:** Visualization, Writing – review & editing. **Pascal Laforêt:** Validation, Writing – review & editing. **Loic Bouilleau:** Visualization, Writing – review & editing. **François Maillot:** Conceptualization, Supervision, Validation, Writing – review & editing.

## Declaration of competing interest

None.

## Data Availability

No data was used for the research described in the article.
